# Nanobody-Displaying Flagellar Nanotubes

**DOI:** 10.1038/s41598-018-22085-3

**Published:** 2018-02-26

**Authors:** Ágnes Klein, Mátyás Kovács, Adél Muskotál, Hajnalka Jankovics, Balázs Tóth, Mihály Pósfai, Ferenc Vonderviszt

**Affiliations:** 1 Bio-Nanosystems Laboratory, Research Institute of Biomolecular and Chemical Engineering, University of Pannonia, Egyetem u. 10, H-8200 Veszprém, Hungary; 2 Department of Earth and Environmental Sciences, University of Pannonia, Egyetem u. 10, H-8200 Veszprém, Hungary; 3 Research Institute for Technical Physics and Materials Science, Hungarian Academy of Sciences, Konkoly Thege u. 29-33, H-1121 Budapest, Hungary

## Abstract

In this work we addressed the problem how to fabricate self-assembling tubular nanostructures displaying target recognition functionalities. Bacterial flagellar filaments, composed of thousands of flagellin subunits, were used as scaffolds to display single-domain antibodies (nanobodies) on their surface. As a representative example, an anti-GFP nanobody was successfully inserted into the middle part of flagellin replacing the hypervariable surface-exposed D3 domain. A novel procedure was developed to select appropriate linkers required for functional internal insertion. Linkers of various lengths and conformational properties were chosen from a linker database and they were randomly attached to both ends of an anti-GFP nanobody to facilitate insertion. Functional fusion constructs capable of forming filaments on the surface of flagellin-deficient host cells were selected by magnetic microparticles covered by target GFP molecules and appropriate linkers were identified. TEM studies revealed that short filaments of 2–900 nm were formed on the cell surface. ITC and fluorescent measurements demonstrated that the fusion protein exhibited high binding affinity towards GFP. Our approach allows the development of functionalized flagellar nanotubes against a variety of important target molecules offering potential applications in biosensorics and bio-nanotechnology.

## Introduction

Functionalized nanotubes are widely used in nanotechnology^1,2^. Fabrication of such nanostructured systems via self-assembly is an attractive approach. Many biological systems have the ability of self-assembly and they can offer a promising starting point to develop new nanomaterials. The bacterial flagellum is an organelle for locomotion which involves a membrane-embedded nanomotor rotating long helical filaments. Flagellar filaments are natural protein nanotubes which have an outer diameter of 23 nm and can grow up to 20 μm^3^. Flagellar filaments can assume various helical and straight forms depending on the environmental conditions^4^. They have a rigid structure which is stable over a wide range of pH. Since their surface properties can be easily modified by genetic engineering techniques or chemical treatments, flagellar filaments are extensively used in nanotechnology as templates for nanoparticle arrays or applied as scaffolds to manufacture nanofibers^5–9^.

Filaments of bacterial flagella are built from thousands of flagellin (FliC) subunits which are also capable of controlled *in vitro* assembly under appropriate conditions^10^. The variable central portion of flagellin has been a favourite target site for insertion of heterologous peptides or small proteins to display them on the cell surface for studying binding interactions and immune responses^11–13^. For example, Lu *et al*. created the FliTrx thioredoxin-flagellin fusion construct by insertion of the *E. coli* thioredoxin protein into the non-essential central region of flagellin^12^. To find an appropriate site for insertion, segments were randomly removed from the variable region of FliC and replaced by the full-length thioredoxin protein. FliTrx variants capable of forming filaments on the bacterial cell surface were selected by immobilized anti-thioredoxin antibodies. In earlier studies foreign segments were quite randomly inserted into the hypervariable region of flagellin causing often structural perturbations which resulted in impaired polymerization behaviour and destabilization of filaments. High-resolution structural studies revealed that the conserved terminal regions of flagellin are involved in subunit interactions in the filament core while the hypervariable central portion of the polypeptide chain, comprising residues 190–284, forms the D3 domain exposed on the filament surface^14,15^. We demonstrated that D3 is a structurally independent part of flagellin which has no direct role in filament formation^16^ and it can be removed or replaced by foreign proteins without influencing polymerization ability^17,18^.

We aim at exploiting the polymerization ability of flagellin to create a variety of building blocks applicable to construct rationally designed multifunctional tubular nanostructures. The concept of our work is to engineer flagellin to give it various functionalities by creating fusions with suitable foreign proteins (like enzymes, binding proteins or reporter units) without adversely affecting polymerization ability. Flagellin-based polymerizable proteins make a novel platform opening the way to fabricate new bionanoassemblies. The prototype of flagellin-based polymerizable proteins has been successfully created by replacing the D3 domain of flagellin with the amino acid sequence of the xylanase A enzyme^17^. A polymerizable GFP variant has been also fabricated which exhibited intensive fluorescence and was capable of efficient filament formation^18^. In these cases, it was relatively simple to construct these fusions because the terminal regions of the inserted proteins were close to each other similarly to the two internal ends of flagellin generated upon removal of D3. However, it is very challenging to construct a functional fusion protein when the two terminal regions of the protein to be inserted are far from each other. In this work a novel procedure has been developed to accomplish internal insertion of foreign proteins into flagellin in a general case. Its applicability is demonstrated by the development of a polymerizable binding protein by creating fusion constructs of flagellin with single-domain antibody (nanobody).

Nanobodies (NBs) are small binding proteins typically comprising of 110–130 residues^19,20^. They are derived by directed evolution from the VHH antigen binding domain of the unique heavy chain antibodies found in *Camelidae*. They share a common β-sheet framework structure. They are available against many important target molecules^21^. NBs offer several advantages compared to conventional antibodies: they are highly stable and soluble, have a strong binding affinity typically in the nanomolar range and can be overexpressed in bacteria. There is an intensely growing number of applications of single-domain antibodies in nanotechnology, nanomedicine and biosensorics^21–24^.

**Figure 1:**
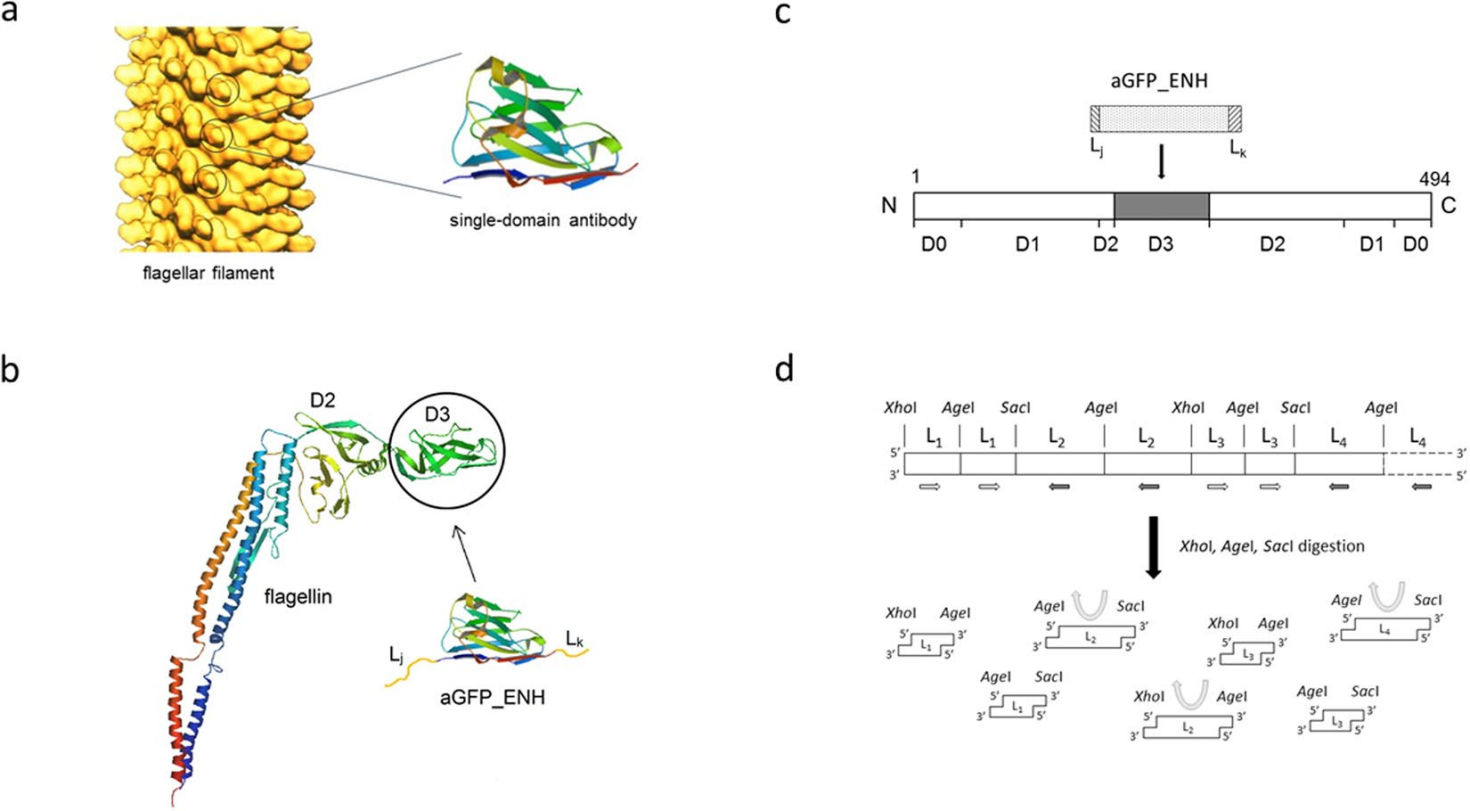
Construction of flagellar nanotubes displaying single-domain antibodies. (**a**) Arrangement of flagellin subunits within the flagellar filament. The outer hypervariable D3 portions (circled) of flagellin subunits, situated on the filament surface, were replaced by single-domain antibodies. The distance between the solvent-exposed nanobodies on the filament surface is about 5.5 nm. Solid surface representation of a longitudinal section of the filament according to Mimori-Kiyosue *et al*. ^35^. (Copyright (1996) National Academy of Sciences, USA.) (**b**) The Ca backbone trace of flagellin (PDB code: 1UCU) and aGFP_ENH nanobody (PDB code: 3K1K). The aGFP_ENH nanobody with various oligopeptide linkers attached to both ends was inserted into the middle part of flagellin to replace the D3 domain. (**c**) Representation of the domain arrangement of flagellin. D0-D2 are discontinuous domains formed by segments from both the N- and C-termini. D3 is constructed by the middle portion of the polypeptide chain consisting of residues 190–283. This internal segment was removed and replaced by a single-domain antibody. (**d**) Oligonucleotide segments coding for the chosen oligopeptide linkers (L_1_, L_2_, …) were joined via segments encoding the cleavage sites of *Xho*I, *Sac*I or *Age*I restriction enzymes to form a multilinker gene. Arrows show the local direction of the reading frame. In the regions indicated by white and black arrows linker segments are coded by the opposite strands of DNA. The multilinker gene was synthesized and fragmented by a mixture of restriction enzymes (*Xho*I, *Sac*I, *Age*I) to obtain linker-encoding segments (shown in the same orientation relative to the reading frame) with sticky ends facilitating construction of the FliC-aGFP_ENH fusion genes (see text).

In order to furnish flagellar nanorods with molecular recognition functionality, we aim to introduce nanobodies into the variable central part of flagellin replacing the D3 domain (Fig. 1a). In this work, we studied formation of functionalized flagellar filaments on the surface of Salmonella cells. As a representative single-domain antibody, we chose the GFP-enhancer nanobody (aGFP_ENH) which can bind GFP and its enhanced and superfolder variants (eGFP, sfGFP) with nanomolar affinity and this interaction leads to a 1.5-fold fluorescence enhancement^25,26^. A large number of flagellin-aGFP_ENH fusion constructs were created applying a variety of linkers and variants capable of forming filaments exhibiting GFP-binding ability were selected and identified. Since different NBs have a very similar scaffold, the selected linkers allow development of a family of flagellin-based, nanotube-forming binding proteins by insertion of various NBs specific for important target molecules.

## Results and Discussion

### Design and gene construction of flagellin-nanobody fusions

In this work, we aim at creating a flagellin variant possessing molecular recognition functionality by replacing the variable D3 domain of flagellin with a single-domain antibody. In order to accomplish functional insertion of a nanobody into the variable central portion of flagellin it is essential to use linkers which allow proper folding of both partners and do not interfere with the polymerization ability of flagellin. Upon removal of the D3 domain the resulting free ends of the D2 domain are separated by about 6 Å^16^. However, according to the available 3D structures of single-domain antibodies (e.g. PDB codes: 3OGO, 3K1K) the N- and C-terminal ends are at the opposite sides of the molecule, far (~38 Å) from each other (see Supplementary Fig. S1). Therefore, it is rather complicated to find appropriate linker segments for insertion into flagellin with computer modelling. Instead, a novel procedure was devised to find suitable linkers which can facilitate construction of well-functioning fusions. In this work the aGFP_ENH nanobody^25^ was used to create the prototype of flagellin-NB fusion proteins.

Altogether 61 oligopeptide segments (L_i_) of different lengths and conformational properties were selected (see Supplementary Table S1), as linker candidates, primarily based on the *linkerdbwww* linker database^27^. The aGFP_ENH nanobody, with linkers from the chosen set randomly attached to its both ends (Fig. 1b-c), was inserted into flagellin replacing the D3 domain. In this way a library of FliC-aGFP_ENH fusion variants was created with a large number (~3700) of randomly paired (L_i_, L_j_) linkers.

The plasmid library coding for the fusion variants was constructed in the following way:

We started with the NT045 pKOT-based plasmid containing the gene of a D3-deleted variant of Salmonella flagellin in which the region coding for the D3 domain was replaced with a short gene cassette containing the recognition sites of *Xho*I, *Age*I, *Xma*I and *Sac*I restriction enzymes to facilitate insertion of foreign genes^17^. Oligonucleotide segments coding for the chosen linkers were designed with restriction enzyme cleavage sites attached to both ends in the following forms: S1-L_i_-S3 and S3-L_i_-S2, where S1, S2 and S3 denote cleavage sites (recognition sequences) for *Xho*I, *Sac*I and *Age*I, respectively. These oligos were joined (via segments encoding the restriction sites) into a multilinker gene (Fig. 1d) which was synthesized. The multilinker gene was fragmented by *Xho*I, *Sac*I and *Age*I enzymes to obtain S1-L_i_-S3 and S3-L_i_-S2 coding segments with sticky ends (see details in Materials and Methods). The gene coding for S3-aGFP_ENH-S3 was synthesized and sticky ends were generated by *Age*I digestion. Linker coding segments were attached to the ends of the aGFP_ENH gene by ligation via the *Age*I sites resulting in S1-L_i_-aGFP_ENH-L_j_-S2 encoding gene constructs which were inserted (ligated) into NT045 plasmids between the *Xho*I and *Sac*I sites. *E. coli* XL10-Gold competent cells were transformed by the plasmid library to facilitate plasmid preparation. About 30% of single colonies contained the insert as judged by colony PCR and sequencing.

### Selection of FliC-aGFP_ENH variants capable of ***in vivo*** filament formation

The plasmid library coding for FliC-aGFP_ENH variants was used to transform flagellin-deficient *SJW2536* *Salmonella* host cells. As a result, a bacterial library was obtained whose members overexpressed flagellin-aGFP_ENH fusions in which the aGFP_ENH nanobody was inserted by randomly paired linkers. Functional fusion constructs were expected to form filaments on the cell surface, each displaying thousands of GFP binding sites. Magnetic microparticles were used to capture cells carrying filaments capable of efficient GFP binding. The following selection procedure was applied:

Transformed cells were grown overnight, centrifuged and washed by PBS. Biotinylated target GFP was added to the cell culture to bind to bacteria with filaments displaying aGFP_ENH nanobodies on their surfaces. Cells with filaments decorated by biotinylated GFP were isolated by streptavidin-coated magnetic microparticles using a magnetic separator. Microparticles were washed several times by PBS to remove nonspecifically (weakly) adsorbed cells. Captured cells were regrown in fresh media and the above selection/wash procedure was repeated 5 times using decreasing amounts of biotinylated GFP to facilitate selection of the strongest binders.

Cells obtained after the selection procedure were cultured in fresh media and distributed on an agar plate containing ampicillin. 10 colonies were randomly chosen, plasmids were isolated and the region coding for the FliC-aGFP_ENH fusion protein was sequenced. The following two linker pairs were identified in 5–5 cases:

1. N-terminal linker: LESAAAATPAVRTVPQYKYAAGVRNTG (L_1N_)
  C-terminal linker: TGRRRMEL (L_1C_)
  and
2. N-terminal linker: LECTATAATAMSAMAQNKDTAG TG (L_2N_)

C-terminal linker: TGRA EL (L_2C_)

(The terminal LE, TG and EL tags originate from the applied *Xho*I, *Age*I and *Sac*I cleavages sites, respectively.) The two variants obtained were designated as FliC-aGFP_ENH_V1 and FliC-aGFP_ENH_V2, respectively.

The fact, that from the ~3700 applied linker pairs only two were detected in the 10 randomly chosen colonies indicated strong selection. In both cases a long N-terminal and a short C-terminal linker were found which meets our expectation based on the available structural information (aGFP_ENH nanobody; PDB code: 3K1K). Since the antigen binding site of the aGFP_ENH nanobody is close to its N-terminus, such asymmetric linkers are required to orient properly the inserted NB to expose its GFP-binding region in the filamentous state. Because NBs share a common structural framework, the identified linker pairs are expected to be applicable to construct other flagellin-NB fusion proteins. As a proof of principle, we inserted a single-domain antibody against *Staphylococcal* enterotoxin B into flagellin using the (L_2N_, L_2C_) linker pair. TEM studies demonstrated that the fusion protein formed short filaments on the surface of SJW2536 host cells (to be published).

### Characterization of filament forming properties

Selected strains carrying flagellar filaments composed of FliC-aGFP_ENH subunits were found to be very weakly motile under the dark-field microscope. This observation indicated that *in vivo* filament formation from FliC-aGFP_ENH subunits was somewhat paralyzed.

**Figure 2:**
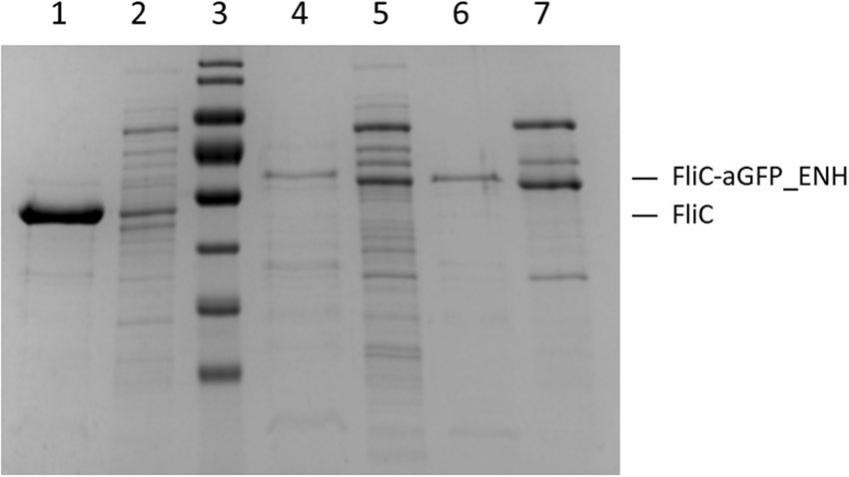
SDS-PAGE analysis of the filament-forming and secreted monomeric fractions of the FliC-aGFP_ENH fusion proteins, overexpressed in flagellin-deficient *SJW2536 Salmonella* host cells. For comparison, similar fractions from cells expressing the D3-deleted flagellin variant (FliC-delD3) were also run on the gel. Lanes 2, 5, 7 are the secreted monomeric fractions for FliC-delD3, FliC-aGFP_ENH_V1 and FliC-aGFP_ENH_V2, respectively. Lanes 1, 4, 6 show the amount of protein subunits for FliC-delD3, FliC-aGFP_ENH_V1 and FliC-aGFP_ENH_V2, respectively, forming filaments on the cell surface. The intensity of the bands suggests that the fusion proteins were mainly secreted into the culture medium and only a smaller fraction formed filaments. The calculated molecular masses of FliC-delD3, FliC-aGFP_ENH_V1 and FliC-aGFP_ENH_V2 are 42.8 kDa, 58.6 kDa and 57.7 kDa, respectively.

The amount of FliC-aGFP_ENH fusion proteins incorporated into filaments on the cell surface was studied by gel electrophoresis (Fig. 2). After centrifugation of the cell cultures, cells were resuspended in PBS and heated to 65 °C to disassemble flagellar filaments which are known to undergo heat-induced depolymerization around 60 °C^28^. Cell bodies were removed by high-speed centrifugation and the supernatant was analysed by SDS-PAGE to visualize the depolymerized FliC-aGFP_ENH fraction. The amount of secreted monomeric subunits was also investigated. Secreted monomers were collected from the cell culture supernatant by TCA precipitation. For comparison, similar fractions from *SJW1103 Salmonella* cells expressing wild-type flagellin were also run on the gel (Fig. 2). The FliC-aGFP_ENH_V1 and FliC-aGFP_ENH_V2 fusion proteins were produced by the cells in significant amounts (lanes 4–5 and 6–7), but they primarily appeared in the cell culture supernatant and only a smaller fraction formed filaments on the cell surface. We found that wild-type flagellin was exported from the cells in a substantially larger amount (lanes 1–2). Since protein translocation by the flagellum-specific export machinery involves unfolding of the exported subunits^29^, decreased export levels of the flagellin-nanobody fusions may reflect the highly stable/resistant beta-sandwich structure of the incorporated nanobody.

It is clearly seen that filament formation was more effective with FliC-aGFP_ENH_V2 than with the FliC-aGFP_ENH_V1 variant (lanes 6 and 4, respectively). Therefore, the V2 variant was used in subsequent investigations for more detailed characterization.

**Figure 3:**
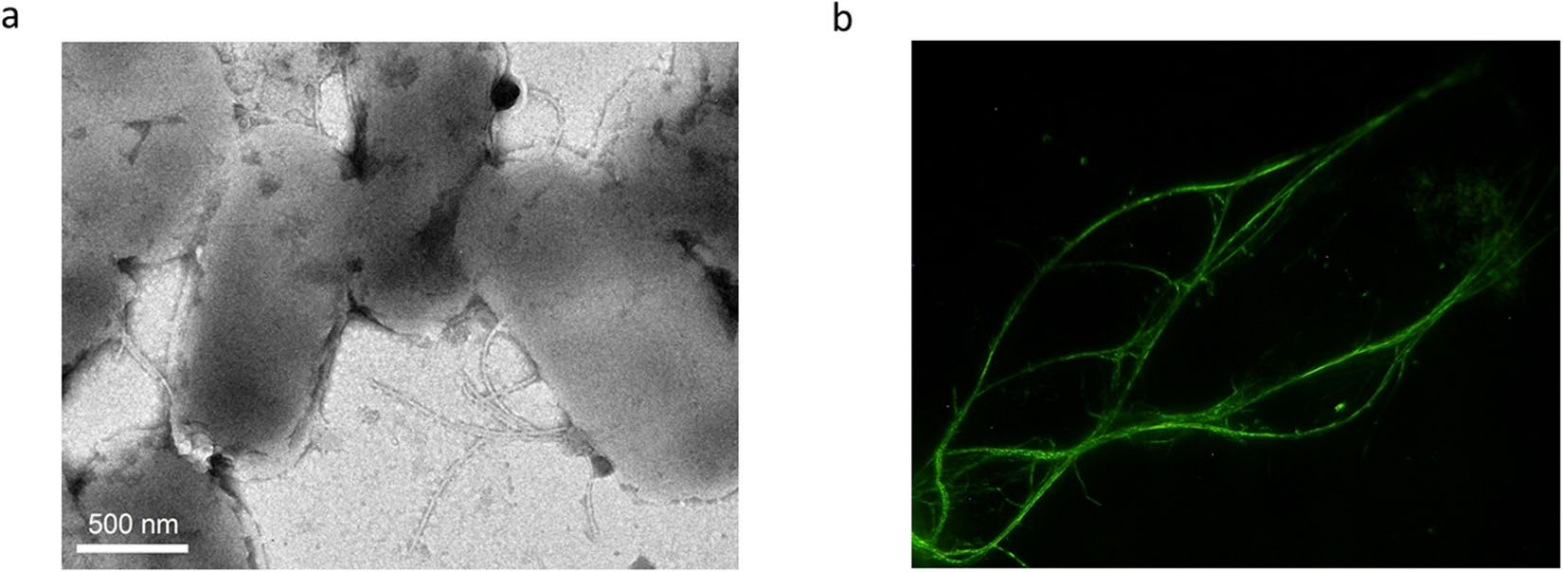
Flagellar nanotube formation from FliC-aGFP_ENH_V2 subunits *in vivo* and *in vitro*. (**a**) Bright-field TEM image of *Salmonella* cells that possess mutant flagellar filaments. Samples were stained with 2% phosphotungstate to enhance image contrast. (**b**) Filaments polymerized from purified subunits as visualized by dark-field optical microscopy. Polymerization experiments were carried out in PBS buffer (pH 7.4) at 2 mg/ml protein concentration and 4 M AS was added to 0.6 M final concentration to initiate filament formation.

First, cells producing FliC-aGFP_ENH_V2 filaments were visualized by transmission electron microscopy (Fig. 3a). In accordance with the SDS-PAGE analysis, mutant subunits produced short filaments on the cell surface. Their length was in the range of 200–900 nm, which is much shorter than the typical length of filaments of wild-type *Salmonella* known to grow filaments as long as 5–15 μm. The observed short filaments explain the poor motility of our mutant bacteria because they are not long enough for efficient helical bundle formation that is required for swimming.

Monomeric FliC-aGFP_ENH subunits were used to reconstruct filaments by ammonium sulphate (AS) precipitation^30^. FliC-aGFP_ENH_V2 readily formed long bundles of filaments upon addition of 0.5 M AS as observed by dark-field microscopy (Fig. 3b). The average length of filaments was controllable by the applied precipitant concentration. Filaments were stable and remained intact for at least several days even after the removal of AS from the solution by spinning down the sample and dissolving the pellet in PBS. Unfortunately, reconstituted filaments showed a strong tendency for aggregation. Further efforts are needed to improve their solubility by modifying their surface charge distribution by applying protein engineering procedures.

It may seem contradictory that polymerization ability of FliC-aGFP_ENH subunits was significantly paralyzed *in vivo*, but they readily assembled into long filaments *in vitro*. However, there are special requirements for efficient filament formation on bacterial cells. Flagellin subunits are synthesized in the cytoplasm and exported by the flagellum-specific export machinery to the site of assembly at the distal end of the filament^29^. They are translocated through the 25 Å wide central channel of the flagellum as partially unfolded monomers and are supposed to acquire their folded conformation in the tiny cavity below the pentameric HAP2 cap attached to the tip of filaments. Efficient filament formation requires compatibility with the export process and fast folding below the HAP2 cap. FliC-aGFP_ENH subunits were primarily found in the cell culture supernatant, indicating that fusion proteins are readily exported by the flagellar export apparatus. Therefore, we think that formation of short filaments reflects slow folding of subunits. At the filament tip, diffusion into the outer culture medium is a competing process with folding and assembly. In the test tube, there are subunits with well-folded D1-D2-aGFP domains and they can readily assemble into long flagellar nanotubes.

### Binding experiments

Selection experiment convincingly demonstrated the GFP-binding ability of filaments formed *in vivo* from flagellin-NB chimeras. However, the strong aggregation of purified or reconstituted FliC-aGFP_ENH_V2 filaments prevented a detailed investigation of these samples. Instead, monomeric subunits were prepared and their GFP-binding ability was further characterized by fluorescence measurements and calorimetric experiments. We aimed to demonstrate that aGFP_ENH can acquire its properly folded structure and retain binding affinity for GFP even upon insertion into the middle part of flagellin.

**Figure 4:**
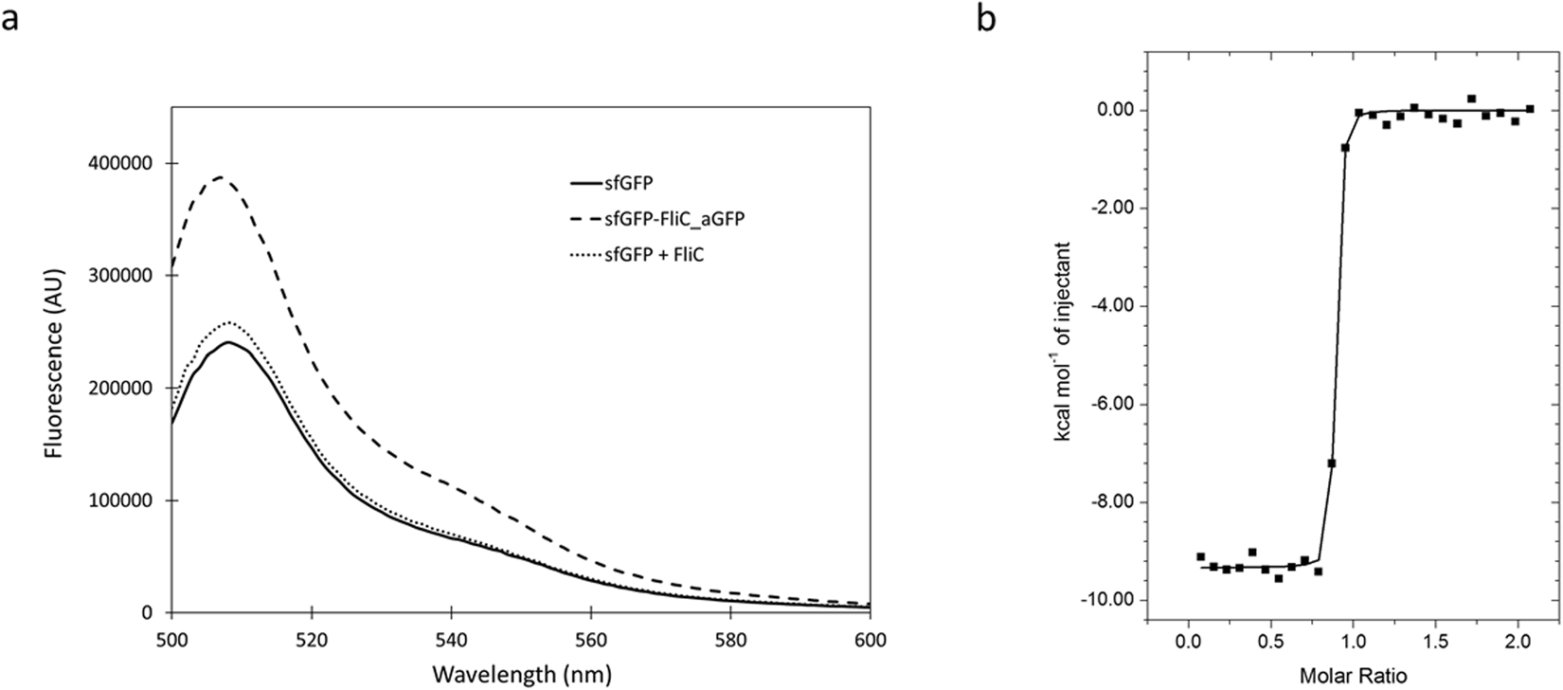
Interaction of the FliC-aGFP_ENH fusion protein with sfGFP. (**a**) The fluorescence emission spectrum of sfGFP (solid) showed an emission maximum at 508 nm. Addition of the FliC-aGFP_ENH fusion protein (dashed) resulted in a nearly 60% increase of fluorescence intensity. As a control experiment, wild-type flagellin was also added (dotted) but it produced only a small effect on sfGFP fluorescence. Measurements were taken in PBS buffer (pH 7.4) at an excitation wavelength of 488 nm. (**b**) Isothermal calorimetric titration of FliC-aGFP_ENH with sfGFP at 25 °C. The FliC-aGFP_ENH sample (c = 0.006 mM) was loaded into the cell and the sfGFP (c = 0.065 mM) solution was injected in 10 μl portions. Changes in binding enthalpy (▪) of the injections are shown as a function of the molar sfGFP to FliC-aGFP_ENH ratio. The solid line is the least-squares fit to the data by using a one-binding-site model, resulting in the following parameters: stoichiometry N = 0.86, dissociation constant K = 1.1·10^−9^ M, binding enthalpy ΔH = −9.3 kcal/mol. Titrations were done in 20 mM Tris-HCl 150 mM NaCl, pH 8.5, buffer.

Interaction of the aGFP_ENH nanobody with eGFP was reported to result in an increase of about 50% in fluorescence intensity^25^. In our experiments superfolder GFP was used which is an eGFP variant^31^ and was expected to produce a fluorescence enhancement effect of a similar magnitude. Fluorescence emission spectra of sfGFP were measured in the presence and absence of FliC-aGFP_ENH_V2 (Fig. 4a). Upon addition of a saturating amount (2-fold molar excess) of FliC-aGFP_ENH, about 58% increase of fluorescence intensity was observed clearly demonstrating the interaction. In control experiments addition of wild-type flagellin to sfGFP resulted only in a small increase (<7%) of fluorescence intensity. The magnitude of fluorescence enhancement caused by FliC-aGFP_ENH binding was similar to that previously obtained for the aGFP_ENH–eGFP interaction^25^, indicating that the aGFP_ENH nanobody preserved its native-like conformation upon insertion into flagellin.

Isothermal titration calorimetric experiments also demonstrated that the FliC-aGFP_ENH_V2 chimera had a strong affinity towards sfGFP (Fig. 4b). The obtained binding curve had a nearly rectangular shape indicating very tight binding. The measured data were analysed by a one-binding-site model. Although, for associations with a very high affinity it is difficult to obtain the equilibrium binding constant by ITC with precision, curve fitting suggested that the dissociation constant characteristic of the interaction was in the nanomolar range. The ΔH enthalpy of binding was 9.3 kcal/M which agreed well with earlier observations concerning the interaction of the aGFP_ENH nanobody with GFP^26^. The stoichiometry of interaction was somewhat lower than 1 (N = 0.86) indicating slight aggregation of the FliC-aGFP_ENH sample or uncertainties in sample concentrations.

Our fluorescence and calorimetric experiments clearly show that the molecular recognition functionality of the aGFP_ENH nanobody is fully preserved upon insertion into the central part of flagellin.

## Conclusions

With the aim to develop binding proteins capable of forming filamentous nanostructures, a fusion construct of flagellin with a single-domain antibody was created. Terminal regions of flagellin are essential for filament formation, however, the variable central part of the polypeptide chain forming the surface-exposed D3 domain offers a promising site for insertion of foreign proteins without disturbing self-assembling ability. In a general case, it is not a trivial task to create a functional fusion protein by internal insertion. It is essential to use suitable linker segments which allow proper functioning of both partners. A novel procedure was devised to find appropriate linkers required for functional insertion. The created FliC-aGFP_ENH fusion protein had self-assembling ability *in vivo* and *in vitro* to form nanotubes displaying thousands of regularly arranged binding sites on their surface. Since single-domain antibodies share a common framework structure, the selected linkers can be used to fabricate other flagellin-based nanobody fusion constructs as well, with a variety of molecular recognition features. This study paves the way for the development of mutant bacterial cells with the ability to recognize specific sites or bind target molecules. As we have recently demonstrated^17,18^, enzymes or reporter proteins can also be fused with flagellin resulting in a plethora of building blocks applicable for functionalized nanotube formation. Flagellin-based fusions proteins with catalytic and molecular recognition functionalities can be assembled to form mixed or block copolymers in a rationally designed way opening new horizons for applications in medical diagnostics, environmental monitoring or bionanotechnology.

## Methods

### Bacterial strains, plasmids and genes

The flagellin-deficient *Salmonella SJW2536* strain was used for selection and overexpression of FliC-aGFP_ENH fusion constructs. The restriction-deficient *Salmonella JR501* strain^32^ was applied to modify plasmids for Salmonella compatibility. A pKOT-based plasmid containing a D3 deletion mutant flagellin (ΔD3_FliC) gene was created as described previously^17^. In this construct the D3 coding region was deleted and replaced by a linker segment containing the recognition sites of *XhoI*, *AgeI*, *XmaI* and *SacI* enzymes. This plasmid served as a cloning vector to create multiple variants of aGFP_ENH inserts in the middle part of the *fliC* gene. The nucleotide sequence of the gene of GFP-enhancer nanobody^25^ was derived from the protein sequence publicly available (PDB accession number: 3K1K). The sequence was optimized for *Salmonella* codon preferences and synthesized with *AgeI* restriction sites at both ends by the Genscript Corporation (Piscataway, NJ, USA).

### Construction of aGFP_ENH insert library

To construct the aGFP_ENH insert library, 61 linkers were selected, mainly from the linkerdbwww linker database^27^, representing various lengths and structures. Each linker coding segment was prepared to be built in on either side of the aGFP_ENH sequence, resulting in 61 different 5′-linkers and 61 different 3′-linkers, altogether 3721 independent linker combinations. To ensure the incorporation in the desired order the 5′-linkers were designed with *XhoI* restriction site on the 5-prime end and *AgeI* site on the 3-prime end, while the 3′-linkers carried *AgeI* site on the 5-prime end and *SacI* site on the 3-prime end. This design determined the order of the insertion during the aGFP_ENH insert library construction resulting in an *XhoI*-3′linker-*AgeI*-aGFP_ENH_gene-*AgeI*-5′linker-*SacI* insertion order.

Oligonucleotides coding for the 61 linkers were grouped into 7 multilinker genes which contained multiple linkers connected through their *AgeI*, *XhoI* or *SacI* restriction enzyme sites (Fig. 1d). The multilinker genes were synthesized and delivered by Genscript Corporation (Piscataway, NJ, USA) cloned into pUC57 plasmids bordered by two *NotI* sites. This design enables the continuous maintenance of our linker set by simple plasmid multiplication in *E.coli* and the single linker-coding segments with sticky ends can be retrieved from the plasmid by simple restriction digestion. For the construction of the aGFP_ENH insert library the multilinker genes were removed from the plasmid by *NotI* digestion then the purified multilinker genes were mixed and digested by *AgeI*. The obtained fragments were ligated onto both ends of the aGFP_ENH gene using *AgeI* restriction sites. The ligation products between 360 and 720 bps were purified from agarose gel using GFX Gel Band Purification kit (GE Life Sciences), digested by *XhoI* and *SacI* enzymes and were inserted into the ΔD3_FliC gene between the *XhoI* and *SacI* restriction sites using the Rapid DNA Ligation Kit (Fermentas). XL10 Gold chemically competent *E.coli* cells (Agilent Technologies) were transformed by the ligation mixture. A small portion of the transformants was spread on agar plates, while the rest were grown in 150 ml LB for plasmid preparation. Following overnight incubation, the transformant colonies on the agar plate were tested by colony hybridization using DIG-labelled aGFP_ENH gene as a probe (Roche) to determine the efficiency of the successful insert incorporation. Moreover, plasmids from randomly selected clones were also sequenced to check the diversity of our library. The purified plasmid mixture was first electroporated into restriction-deficient *JR501 Salmonella* strain to produce plasmids for Salmonella compatibility. These plasmids were then electroporated into the FliC-deficient *SJW2536 Salmonella* strain for the subsequent panning and selection protocol.

### Panning and selection

*SJW2536 Salmonella* cells transformed by the aGFP_ENH insert library were grown overnight. The cells were harvested by centrifugation and resuspended in blocking solution containing 1xPBS, 1% nonfat milk and 1% mannose. The test tubes used for the selection procedure were also pretreated by blocking solution to prevent nonspecific binding to the tube wall. Biotinylated sfGFP was added to the bacterial cell suspension and incubated at 4 °C for 1 hour to develop GFP – FliC-aGFP_ENH interactions. In the next step 40 μl of SiMAG-Streptavidine magnetic microparticle (size: 1 µm, c = 10 mg/ml; Chemicell GmbH) was added and incubated at 4 °C for 30 minutes to form biotin-streptavidin bonds. Following separation by a MagnetoPURE magnetic separator (Chemicell GmbH) the magnetic microparticles were washed 10 times using blocking buffer and the bound bacterial cells were removed from the surface of the particles by vigorous vortexing. At the end of the first cycle the selected bacteria were grown overnight to form the next population for the second selection cycle. The selection procedure was repeated 5 times.

Selected cells were visualized by electron microscopy with a JEOL JEM 3010 instrument, operated at 300 kV accelerating voltage. Samples were deposited onto Cu TEM grids covered by a Formvar/carbon film and negatively stained with 2% phosphotungstate (pH 7.0) to enhance image contrast.

### Protein expression and purification

C-terminally His-tagged superfolder GFP (sfGFP) was produced and isolated according to Klein *et al*.^18^ Collected fractions were dialyzed against PBS and the concentration was set to 2 mg/ml. Purified sfGFP was biotinylated using EZ-link Sulpho-NHS-LC-LC Biotin (Thermo Scientific) following the manufacturer’s instructions.

Purification of the FliC-aGFP_ENH fusion protein was performed as follows: the bacterial pellet from 1 L overnight culture (at 37 °C in 3% yeast extract) of the selected cell line was collected by centrifugation at 4000 g for 30 min and suspended in 5 ml PBS. The sample was heated to 65 °C for 10 minutes to depolymerize flagellar filaments. Cell bodies and aggregates were removed by high-speed centrifugation at 70.000 rpm for 30 minutes using a MLA80 rotor with an Optima MAX-E (Beckman Coulter) ultracentrifuge. The supernatant was precipitated by 1.2 M ammonium sulphate and incubated at 25 °C overnight. The precipitated fraction was obtained by centrifugation at 70.000 rpm for 30 minutes and dissolved in 3 ml PBS. Insoluble material was removed by another run of high-speed centrifugation and the supernatant fraction contained FliC-aGFP_ENH in monomeric form.

Protein concentrations of FliC-aGFP_ENH samples were determined from absorption measurements at 280 nm using a molar extinction coefficient of ε_280_ = 3.75 × 10^4^ M^−1^ cm^−1^ calculated from the known aromatic amino acid content of the fusion protein^33^. To calculate the concentration of sfGFP solutions from the absorbance at 488 nm, the extinction coefficient known for eGFP (ε_488_ = 5.6 × 10^4^ M^−1^ cm^−1^) was used. Superfolder GFP is an eGFP variant and the absorption spectra of the two proteins in the visible-UV region were reported to be virtually the same^34^. Purity of protein samples was checked using SDS-PAGE followed by Coomassie blue R-250 staining.

### Polymerization experiments

The polymerizing ability of the FliC-aGFP_ENH fusion protein was investigated by inducing polymerization by ammonium sulphate (AS)^30^. Protein solutions of ~2 mg/ml were prepared in PBS (pH 7.4) and 4 M AS was added to various final concentrations in the range of 0.4 M to 0.8 M. Filament formation was observed after 24 h of incubation at 25 °C. Filaments were observed by dark-field optical microscopy with an Olympus BX50 microscope.

### Fluorescence measurements

Experiments were performed with a Fluoromax-2 (Jobin-Yvon) fluorescence spectrophotometer. The emission spectrum of sfGFP was measured applying excitation at 488 nm and recording fluorescence intensity in the 500–600 nm wavelength range. All measurements were carried out in PBS buffer (pH 7.4). The concentration of sfGFP was 1 μg/ml in the cell. The FliC-aGFP_ENH_V2 fusion protein or wild-type flagellin was added in 2-fold molar excess.

### Calorimetric studies

ITC experiments were carried out with a VP-ITC titration calorimeter (MicroCal, Northampton, MA). Measurements were done in 20 mM Tris-HCl buffer containing 150 mM NaCl (pH 8.5). Protein samples were extensively dialyzed against the buffer at 4 °C. All solutions were thoroughly degassed before use by stirring under vacuum. The FliC-aGFP_ENH solution was loaded into the calorimetric cell (c = 0.06 mM) and 10 μl portions of concentrated sfGFP solution (c = 0.065 mM) were injected. As a control experiment, the sfGFP solution was also injected into the buffer and the measured heats of dilution were subtracted from the main experiment. Calorimetric data were analysed using MicroCal Origin software fitting them by a single-binding-site model.

### Data availability

The datasets generated during the current study are available from the corresponding author on reasonable request.

## Electronic supplementary material

Supplementary table and figure
