# Supplementary material for: Nanobody-Displaying Flagellar Nanotubes
Source: Sci Rep. 2018 Feb 26;8:3584. doi: 10.1038/s41598-018-22085-3 (PMC5832153; doi:10.1038/s41598-018-22085-3)
Supplement: Supplementary file 1 — Supplementary table and figure [file 41598_2018_22085_MOESM1_ESM.pdf]

Nanobody-Displaying Flagellar Nanotubes

Ágnes Klein[1], Mátyás Kovács[1], Adél Muskotál[1], Hajnalka Jankovics[1], Balázs Tóth[1], Mihály Pósfai[2], Ferenc Vonderviszt[1,3*]

[1]*Bio-Nanosystems Laboratory, Research Institute of Biomolecular and Chemical Engineering, University of Pannonia, Egyetem u. 10, H-8200 Veszprém, Hungary*

[2]*Department of Earth and Environmental Sciences, University of Pannonia, Egyetem u. 10, H-8200 Veszprém, Hungary*

[3]*Research Institute for Technical Physics and Materials Science, Hungarian Academy of Sciences, Konkoly Thege u. 29-33, H-1121 Budapest, Hungary*

[*]*Corresponding author:* Tel: +36-88-624974 Fax: +36-88-428275

E-mail address: von007@almos.uni-pannon.hu

	Linkers
GROUP 1	VQQKYKVSDTAATVTG<br>LEPRVFREVTVKGI<br>VIGYMPIPVGVA<br>FSSQKGSVGYA<br>AKIYAELVGFG<br>LSGEYDKAV<br>VSKGYEKA<br>TGEQGLG<br>LPEEKG<br>ALEG<br>GSGM<br>NVG
GROUP 2	**GVTPSTTALPDIVNLSTNYLDKNTREDRIHSIKDF**<br>GVTPSTTALPDIVNLSTNYLDKNTREDRIH<br>GVTPSTTALPDIVNLSTNYLDKNTRE<br>GVTPSTTALPDIVNLSTNYLDKN<br>GVTPSTTALPDIVNLSTNYL<br>GVTPSTTALPDIVNLSTN<br>GVTPSTTALPDIVNLS<br>GVTPSTTALPDIV<br>GVTPSTTA<br>GVTPST<br>GVTPS<br>GVT<br>GV
GROUP 3	**SAAAATPAVRTVPQYKYAAGVRNPQQHLNAQPQVTMQQPAVHVQGQEPL**<br>SAAAATPAVRTVPQYKYAAGVRNPQQHLNAQPQVTMQQPAVHV<br>SAAAATPAVRTVPQYKYAAGVRNPQQHLNAQPQVTMQQPA<br>SAAAATPAVRTVPQYKYAAGVRNPQQHLNAQPQV<br>SAAAATPAVRTVPQYKYAAGVRNPQQHLNA<br>SAAAATPAVRTVPQYKYAAGVRNPQQ<br>SAAAATPAVRTVPQYKYAAGVRN<br>SAAAATPAVRTVPQYKYAAG<br>SAAAATPAVRTVPQYKYA<br>SAAAATPAVRTVPQY<br>SAAAATPAVRTV<br>SAAAATPAV<br>SAAAAT<br>SAAA<br>SAA<br>SA
GROUP 4	GTTTTATTMGAMTQNENTAGMGNAQQHLHAQTQMAVQQTAMHV<br>CTATAATAVGAVAQDKNTAGVRHTQQHLNTQAQM<br>GMATRAATLTDIMNLGAHNLDKNAREDGIH<br>CTATAATAVCAMTQDEDTARVRHAQQ<br>RMTSGAATLAYIMHLGANNLNEH<br>CTATAATAMSAMAQNKDTAG<br>SVTASAATLANVMHLRAN<br>STATAATAVRAMTQD<br>LKAGVFGEVAVKGV<br>RMAARAATLTNIV<br>TEIDAKLVGFR<br>CTATAATAM<br>LGREDDETV<br>AREQGLR<br>GVATRA<br>RRRM<br>RTAT<br>DMR<br>RVA<br>RA

Supplementary Table S1.

Linker segments used for the insertion of the aGFP_ENH single-domain antibody into the variable middle part of flagellin. Group1 contains linkers of various lengths and conformational properties selected from the linkerdbwww linker database (http://www.ibi.vu.nl/programs/linkerdbwww/). Linkers in Group2 and Group3 are derived by successive truncation of two long linkers (shown in bold) obtained from the linkerdbwww database. Random oligopeptides (Group4) were also applied as potential linker segments.

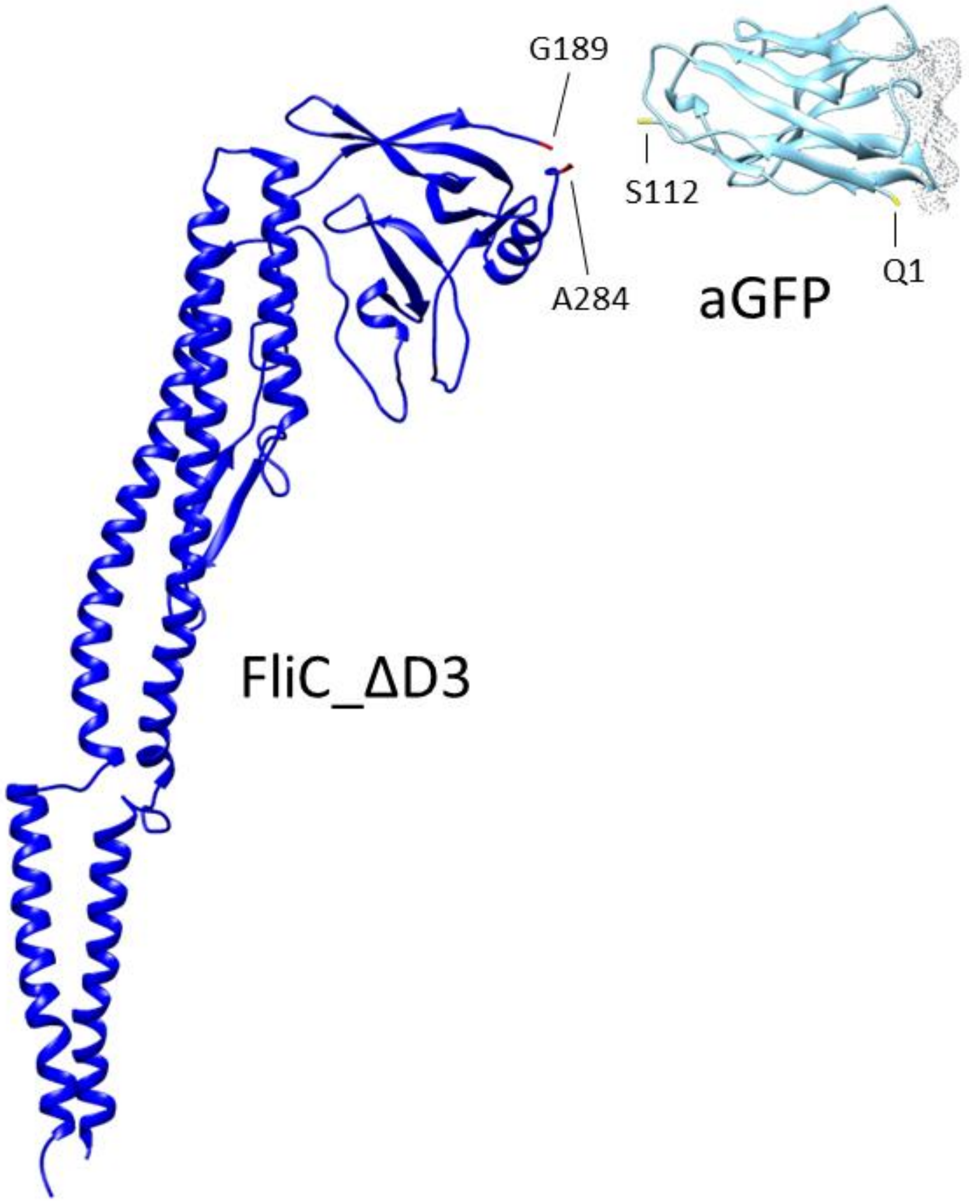

Supplementary Figure S1.

The polypeptide backbone of flagellin (blue) after removal of the D3 domain (FliC_ΔD3). The resulting free ends of the D2 domain (G189 and A284) are close to each other, separated by about 6 Å. The N- and C-terminal ends of the aGFP single domain antibody (light blue) are at the opposite sides of the molecule. Suitable linker segments are required for functional insertion of aGFP into the D3 deleted flagellin which allow proper orientation of the GFP-binding region (indicated by a dotted surface) of the aGFP nanobody. Molecular modelling was done by the UCSF Chimera program (1).

Reference

1. Pettersen, E. F., Goddard, T. D., Huang, C. C., Couch, G. S., Greenblatt, D. M., Meng, E. C. & Ferrin, T. E. UCSF Chimera – a visualization system for exploratory research and analysis. *J. Comput. Chem.* **13**, 1605-1612 (2004).
